# Myeloid A20 is critical for alternative macrophage polarization and type-2 immune-mediated helminth resistance

**DOI:** 10.3389/fimmu.2024.1373745

**Published:** 2024-04-12

**Authors:** Ioanna Petta, Marie Thorp, Maarten Ciers, Gillian Blancke, Louis Boon, Tim Meese, Filip Van Nieuwerburgh, Andy Wullaert, Richard Grencis, Dirk Elewaut, Geert van Loo, Lars Vereecke

**Affiliations:** ^1^ Department of Internal Medicine and Pediatrics, Ghent University, Ghent, Belgium; ^2^ VIB Center for Inflammation Research, Ghent, Belgium; ^3^ JJP Biologics, Warsaw, Poland; ^4^ Laboratory of Pharmaceutical Biotechnology, Ghent University, Ghent, Belgium; ^5^ NXTGNT, Ghent University, Ghent, Belgium; ^6^ Cell Death Signaling Lab, Department of Biomedical Sciences, University of Antwerp, Antwerp, Belgium; ^7^ Lydia Becker Institute of Immunology and Inflammation, Wellcome Centre for Cell Matrix Research, School of Biological Sciences, Faculty of Biology, Medicine and Health, The University of Manchester, Manchester, United Kingdom; ^8^ Department of Biomedical Molecular Biology, Ghent University, Ghent, Belgium

**Keywords:** immunity to parasites, helminth infection, innate immunity, adaptive immunity, type-2 response, macrophage polarization, A20 (TNFAIP3), intestinal immunity

## Abstract

**Background:**

Protective immunity against intestinal helminths requires induction of robust type-2 immunity orchestrated by various cellular and soluble effectors which promote goblet cell hyperplasia, mucus production, epithelial proliferation, and smooth muscle contractions to expel worms and re-establish immune homeostasis. Conversely, defects in type-2 immunity result in ineffective helminth clearance, persistent infection, and inflammation. Macrophages are highly plastic cells that acquire an alternatively activated state during helminth infection, but they were previously shown to be dispensable for resistance to *Trichuris muris* infection.

**Methods:**

We use the in vivo mouse model A20myel-KO, characterized by the deletion of the potent anti-inflammatory factor A20 (TNFAIP3) specifically in the myeloid cells, the excessive type-1 cytokine production, and the development of spontaneous arthritis. We infect A20^myel-KO^ mice with the gastrointestinal helminth *Trichuris muris* and we analyzed the innate and adaptive responses. We performed RNA sequencing on sorted myeloid cells to investigate the role of A20 on macrophage polarization and type-2 immunity. Moreover, we assess in A20^myel-KO^ mice the pharmacological inhibition of type-1 cytokine pathways on helminth clearance and the infection with *Salmonella typhimurium*.

**Results:**

We show that proper macrophage polarization is essential for helminth clearance, and we identify A20 as an essential myeloid factor for the induction of type-2 immune responses against *Trichuris muris*. A20^myel-KO^ mice are characterized by persistent *Trichuris muris* infection and intestinal inflammation. Myeloid A20 deficiency induces strong classical macrophage polarization which impedes anti-helminth type-2 immune activation; however, it promotes detrimental Th1/Th17 responses. Antibody-mediated neutralization of the type-1 cytokines IFN-γ, IL-18, and IL-12 prevents myeloid-orchestrated Th1 polarization and re-establishes type-2-mediated protective immunity against *T. muris* in A20^myel-KO^ mice. In contrast, the strong Th1-biased immunity in A20^myel-KO^ mice offers protection against *Salmonella typhimurium* infection.

**Conclusions:**

We hereby identify A20 as a critical myeloid factor for correct macrophage polarization and appropriate adaptive mucosal immunity in response to helminth and enteric bacterial infection.

## 1 Introduction

Infections with parasites affect more than a quarter of the world’s population and are most prevalent in developing countries with poor hygiene and sanitation (1). The soil-transmitted helminths *Ascaris lumbricoides*, *Trichuris trichiura*, *Ancylostoma duodenale*, and *Necator americanus* are responsible for most infections in humans and can cause severe morbidity (2). In order to study immunity against helminth infections, most studies are done using the murine parasite *Trichuris muris*, a representative model for the human helminth *Trichuris trichiura*, which infects 465 million people worldwide (3). Protective immunity against helminths involves complex host–parasite interactions and is characterized by a robust type-2 (Th2) response, orchestrated both on the immune and intestinal epithelial cell levels. Epithelial cells, the first host cells to encounter the parasite, secrete IL-25, IL-33, and TSLP, which lead to activation and significant expansion of innate immune cells including macrophages, eosinophils, basophils, and mast cells. Balanced innate immune activation primes anti-helminth adaptive Th2 immunity (4). Th2 cells secrete high levels of IL-4, IL-5, and IL-13; promote the production of helminth-specific IgG immunoglobulins by plasma cells, goblet cell hyperplasia, mucus production, epithelial proliferation, and intestinal peristalsis to expel worms; and re-establish immune homeostasis. In contrast, susceptibility to parasite infection is associated with T helper type-1 (Th1) response, characterized by increased secretion of Th1 cytokines such as IFN-γ, IL-12, and IL-18, which antagonize type-2 immunity and favor chronic helminth infection.

The role of macrophages in parasite clearance is controversial. Macrophages have been shown to regulate anti-helminth immunity, as they can trap and kill parasite larvae, but they also contribute to tissue repair and the resolution of type-2 inflammation. Upon helminth infection, macrophages adopt an “alternatively activated” macrophage phenotype (M2-like), which involves the production of various effector molecules including Arginase-1 (Arg1) and Resistin-like protein alpha/beta (Relmα/β), which both have been shown to have an elementary role in regulating Th2 response but also in limiting type-2 immunopathology (5). In contrast, macrophages have been shown to be dispensable for parasite expulsion. More specifically, *in-vivo* pharmacological inhibition of Arg1 or mice deficient in Arg1 are still able to induce effective type-2 immunity and clear *T. muris* infection (6). Moreover, depletion of macrophages by clodronate-loaded liposomes does not prevent the induction of protective type-2 immunity and *T. muris* expulsion (7).

A20 (also known as tumor necrosis factor-induced protein 3, TNFAIP3) is a negative regulator of various inflammatory signaling pathways induced by various pattern recognition receptors (PRRs, including TLRs and NLRs), cytokine receptors (IL-1β, TNF), and T-cell receptors. The anti-inflammatory properties of A20 are commonly attributed to its ability to suppress inflammatory NF-κB signaling, but recent studies in mice have demonstrated that A20 primarily suppresses inflammation by preventing necroptotic cell death (8, 9). A20 exerts its function by acting as a ubiquitin-editing and ubiquitin-binding protein, thereby interfering with downstream signaling events (10, 11). Myeloid-specific A20 deficiency in mice (A20^myel-KO^) leads to the development of spontaneous arthritis, which is characterized by hyperactive NLRP3 inflammasome activity and excessive inflammatory type-1 cytokine production (12). Macrophages lacking A20 have been shown to secrete *in-vivo* increased levels of IL-1β, TNF, IL-6, and IL-18, due to aberrant TLR signaling activation, creating a potent pro-inflammatory environment (13). Moreover, it was recently shown that A20 in macrophages plays a crucial role in lung pathology by regulating the IL-33-mediated STAT1 signaling, which in turn leads to increased IFN-γ secretion by macrophages (14). In the context of airway allergy, the protective effects of A20 were observed upon its deletion in lung epithelial cells, dendritic cells, and mast cells (15, 16). Myeloid A20 is also important for protection against influenza virus infection, in an IFN-α/β-dependent manner (17). We previously reported that deletion of A20 in intestinal epithelial cells does not lead to spontaneous intestinal inflammation but leads to increased sensitivity to DSS colitis and TNF toxicity. Moreover, despite the pro-inflammatory phenotype of A20^myel-KO^ mice, myeloid A20 deficiency does not induce spontaneous intestinal inflammation, unless A20 is also deleted in intestinal epithelial cells (18). Overall, A20 has been studied in an array of inflammatory, autoimmune, allergic, and viral infection models, but its role during gastrointestinal helminth infection and type-2-mediated worm clearance has never been assessed. Considering the multifaceted roles of A20 in modulating diverse inflammatory and cell death pathways, we herein explore the significance of A20 expression within myeloid cells for managing anti-helminthic immune responses. To this end, we employed myeloid-specific A20 knockout mice with the *T. muris* helminth infection model. In this study, we investigate for the first time the role of myeloid A20 in intestinal mucosal immunity and in the context of helminth (*T. muris*) and bacterial (*S. typhimurium*) infection. We report that myeloid A20 deficiency leads to the expansion of myeloid cells and sensitivity to infection with *T. muris*. A20 deletion is associated with strong classical or M1 polarization of macrophages which prevents type-2 immune polarization in an IFN-γ, IL-12, and IL-18-dependent manner. In contrast, we show that the enhanced Th1 response in A20^myel-KO^ mice protects against infection with *S. typhimurium*. In this study, we identify A20 as a critical rheostat for macrophage polarization during infection, which balances type-2 and type-1 adaptive immune responses during helminth and enteric bacterial infection, respectively.

## 2 Results

### 2.1 Myeloid A20 deficiency results in altered intestinal immune cell composition

**Figure 1:**
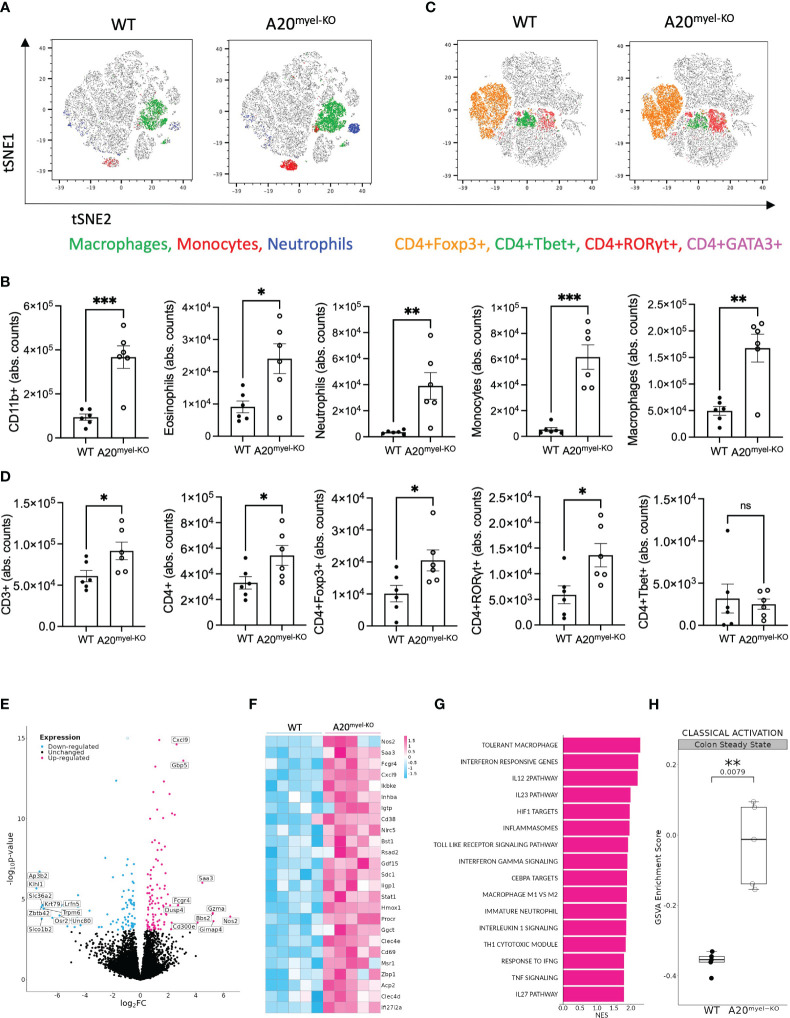
A20 deletion in myeloid cells affects immune cell composition and favors M1 macrophage polarization. **(A, B)** Flow cytometry analysis of colon lamina propria of WT (*n* = 6) and A20^myel-KO^ (*n* = 6) mice for the myeloid and **(C, D)** T-cell composition, presented in t-SNE plots (upper panels) and bar graphs (lower panels). All data are presented as means ± s.e.m. Statistical significance was determined by a two-sided Student’s *t*-test. **(E)** Bulk RNA sequencing volcano plots presenting the differentially expressed genes in CD11b^+^ sorted cells of WT (*n* = 5, blue) and A20^myel-KO^ (*n* = 5, magenta) colon samples. **(F)** Heatmap showing the top 25 M1 markers to be upregulated in A20^myel-KO^ colon compared with WT. **(G)** Gene ontology pathway analysis showing the positively regulated pathways in A20^meyl-KO^ colon CD11b^+^ cells. **(H)** Gene scoring analysis using an M1 signature consisting of 700 genes to compare the expression in WT and A20^myel-KO^ colon. * p<0,05, ** p<0,01, *** p<0,001. Ns, Not significant.

Myeloid-specific A20-deficient mice (A20^myel-KO^) develop spontaneous arthritis and show signs of systemic inflammation, including splenomegaly (12). Despite their pro-inflammatory phenotype, A20^myel-KO^ mice do not develop spontaneous intestinal inflammation (**Supplementary Figures 1A–C**), as previously reported (18). To study intestinal mucosal immunity in more detail, we performed immune phenotyping of the colonic lamina propria in A20^myel-KO^ and wild-type (WT) littermate control mice by flow cytometry and showed that myeloid-specific A20 deletion leads to the expansion of various myeloid immune cell subsets, including eosinophils, neutrophils, monocytes, and macrophages, compared with WT littermate controls (**Figures 1A, B**). In addition, colonic lamina propria T-cell subsets in A20^myel-KO^ mice are characterized by increased infiltration of CD3^+^ and CD4^+^ T cells and, more specifically, by an expansion of Th17 (CD4^+^RORγt^+^) and T regulatory (CD4^+^Foxp3^+^) cells (**Figures 1C, D**). The lack of spontaneous inflammation in the colon, despite increased immune cell infiltration, can be attributed to the high levels of immunosuppressive T regulatory cells. Similar immune cell composition is also observed in A20^myel-KO^ spleens (**Supplementary Figures 1D–K**). In order to obtain more insights into myeloid immune cell function, we performed bulk RNA sequencing on sorted myeloid cells (CD11b^+^) from colon lamina propria (**Figures 1E–H**; **Supplementary Figure 2F**) and joint (combined ankle and knee synovium) tissue (**Supplementary Figures 2A–E**) from A20^myel-KO^ and WT littermates in steady state. The top upregulated genes in colonic A20^myel-KO^ CD11b^+^ cells are involved in IFN signaling and classical macrophage differentiation (M1), including *Cxcl9*, *Gbp5* (19), *Saa3*, *Gzma*, and *Nos2 (* 20) (**Figures 1E, F**). Gene Ontology (GO) pathway analysis revealed that the expression profiles of colonic myeloid cells of A20^myel-KO^ mice are associated with macrophage tolerance and cytokine pathways including IL-12, IL-23, IL-22, IL-17, IFN-γ, and TLR4 signaling (**Figure 1G**). Gene scoring analysis using an “M1 signature calculation” (21) indeed showed a significantly higher M1 score in the A20^myel-KO^ colonic myeloid compartment compared with WT colonic myeloid cells (**Figure 1H**). RA patients show an enhanced M1/Th1 profile which is characterized by increased TLR signaling and secretion of inflammatory cytokines such as TNF, IL-1β, IL-18, and IFN-γ (22). Accordingly, analysis of A20^myel-KO^ synovial myeloid cells (CD11b^+^) revealed transcriptional upregulation of M1-related genes, including different guanylate-binding protein (Gbp) family members (typically induced by IFN-γ) (23), IFN-γ-inducible GTPases (*Gm4841*) (24), acute phase response markers (such as Saa3) (25), and TLR1 pathway genes (26). Moreover, GO pathway analysis revealed that positively regulated pathways in the synovial myeloid compartment of A20^myel-KO^ mice include IFN signaling, proinflammatory cytokine signaling (IL-1, IL-6, IL-17, IL-23), NF-κB and inflammasome signaling, along with a significantly upregulated M1 gene signature (**Supplementary Figures 2A–E**). Together, these data indicate that A20 deletion in myeloid cells induces M1 priming in both intestinal and synovial macrophages.

### 2.2 Myeloid A20 is essential for protective immunity against *Trichuris muris* infection

**Figure 2:**
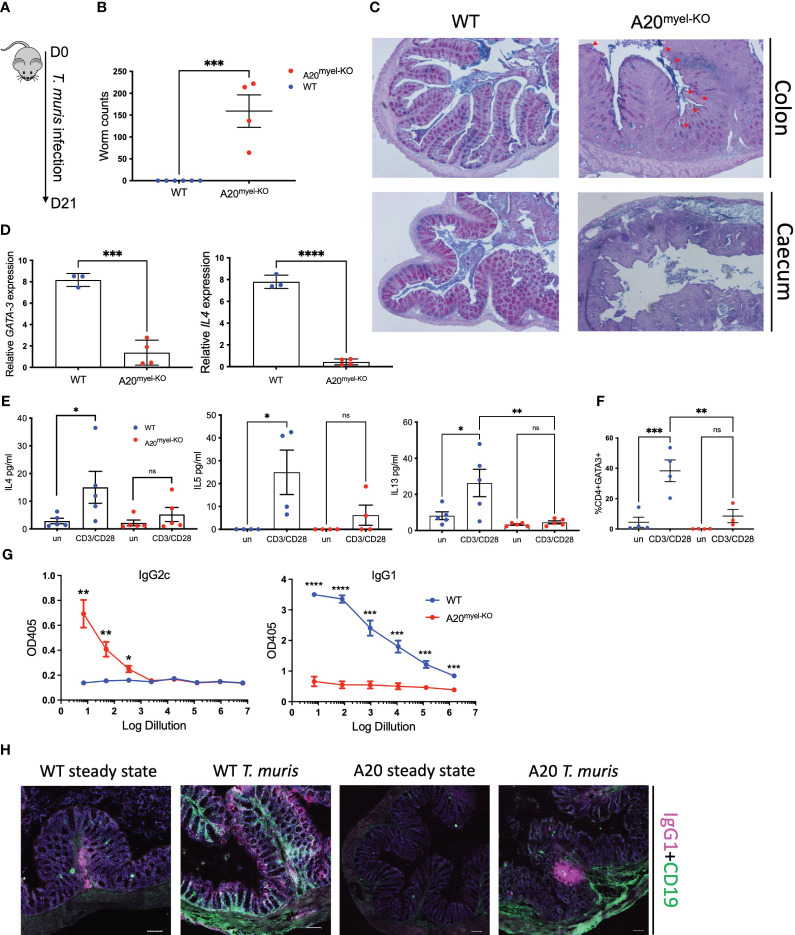
A20^myel-KO^ mice are susceptible to *Trichuris muris* infection. **(A)** Infection scheme with *Trichuris muris* gastrointestinal helminth. **(B)** Worm counts in the cecum of WT (*n* = 6, blue) and A20^myel-KO^ (*n* = 4, red) 21 days after infection with *T. muris*. **(C)** Representative images of AB/PAS staining of WT and A20^myel-KO^ colon (upper panel) and cecum (lower panel) 21 days after *T. muris* infection. Scale bars 100 μm. **(D)** GATA3 and IL-4 relative expression in WT (*n* = 3) and A20^myel-KO^ (*n* = 4) total colon lysates. **(E)** Cytokine levels of IL-4, IL-5, and IL-13 in the supernatant restimulated with a-CD3/a-CD28 WT and A20^myel-KO^ mLN for 48 h. **(F)** CD4^+^GATA3^+^ levels determined by flow cytometry analysis in restimulated mLN and of WT and A20^myel-KO^. **(G)** ELISA for the measurement of IgG2c and IgG1 levels in the blood serum of WT and A20^myel-KO^ mice infected with *T. muris* for 21 days. **(H)** MACSima imaging for IgG1 (magenta) and CD19 (green) of WT and A20^myel-KO^ colon sections at steady state and upon infection with *T. muris* for 21 days. Scale bars 100 μm. All data are presented as means ± s.e.m. Statistical significance was determined by a two-sided Student’s *t*-test or by one-way ANOVA for multiple comparisons. * p<0,05, ** p<0,01, *** p<0,001, **** p < 0.0001. Ns, Not significant.

**Figure 3:**
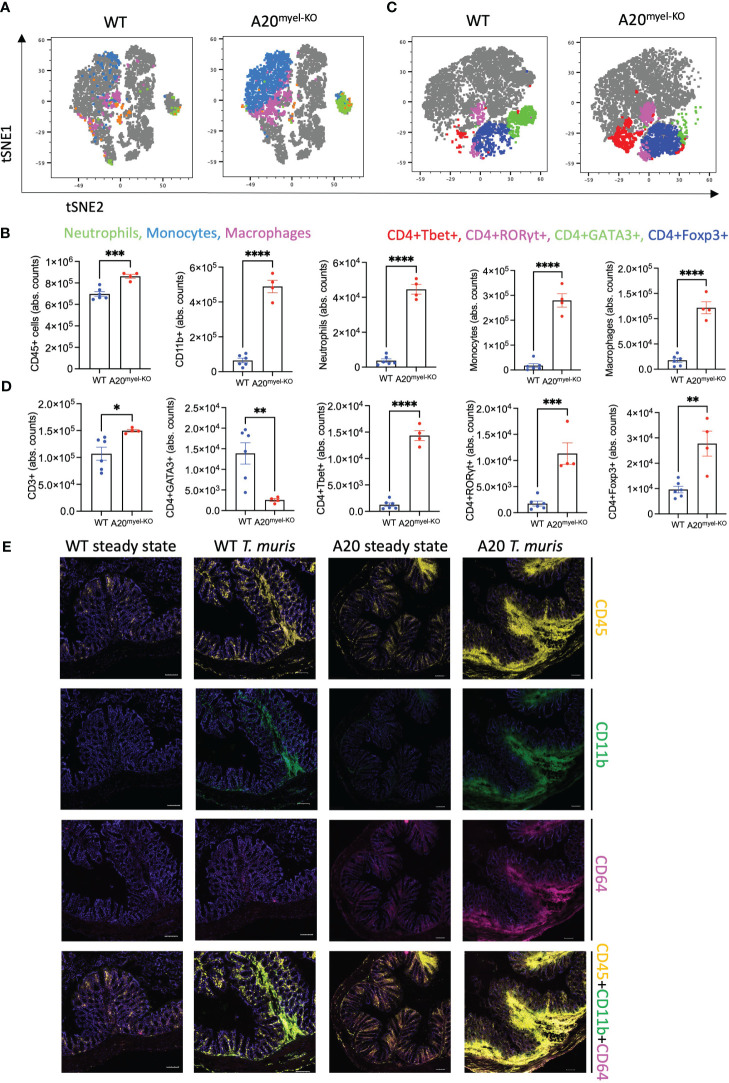
Deletion of A20 in myeloid cells hampers Th2 immunity and promotes Th1 response upon *Trichuris muris* infection. **(A, B)** Flow cytometry analysis of colon lamina propria of WT (*n* = 6) and A20^myel-KO^ (*n* = 4) mice infected with *T. muris* for the myeloid and **(C, D)** T-cell composition, presented in t-SNE plots (upper panels) and bar graphs (lower panels). **(E)** MACSima fluorescent imaging for CD45 (yellow), CD11b (green), and CD64 (magenta) of WT and A20^myel-KO^ colon sections at steady state and upon infection with *T. muris* for 21 days. Scale bar 100 μm. * p<0,05, ** p<0,01, *** p<0,001, **** p < 0.0001.

**Figure 4:**
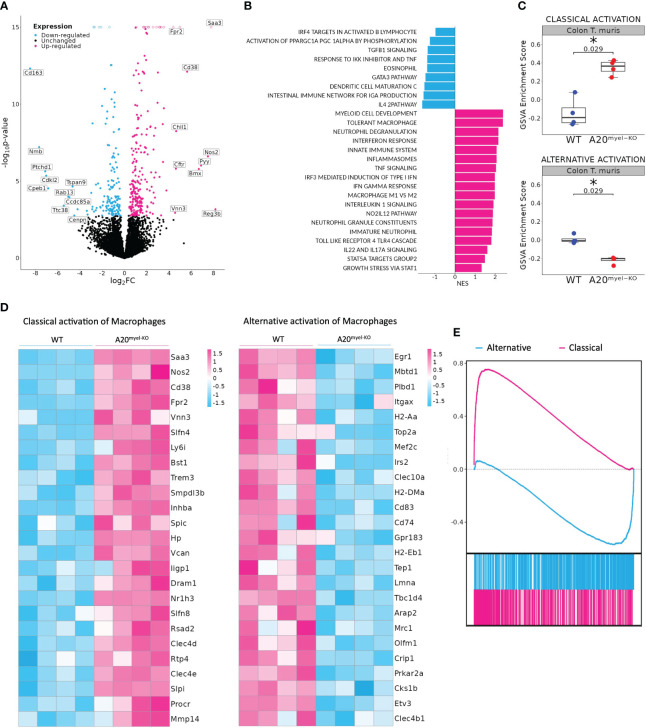
A20^myel-KO^ are characterized by M1 macrophage signature upon *Trichuris muris* infection. **(A)** Bulk RNA sequencing volcano plots presenting the differentially expressed genes in CD11b^+^ sorted cells of WT (blue) and A20^myel-KO^ (magenta) colon samples of mice infected with *T. muris* for 10 days. **(B)** Gene ontology pathway analysis showing the positively regulated pathways in WT (blue) and A20^meyl-KO^ (magenta) colon CD11b^+^ cells of mice infected with *T. muris* for 10 days. **(C)** Gene scoring analysis using M1 and M2 signatures, consisting of 710 and 806 genes, respectively, to compare the expression in WT and A20^myel-KO^ colon upon *T. muris* infection. **(D)** Heatmap showing the upregulation of the top 25 markers related to classical activation of macrophages (M1) in A20^myel-KO^ mice compared with WT (left). Heatmap showing the upregulation of the top 25 markers related to the alternative activation of macrophages (M2) in WT compared with A20^myel-KO^ mice (right). **(E)** Gene set enrichment analysis (GSEA) plots visualizing the enrichment of classical (M1, upper panel) and alternative (M2, lower panel) activation of macrophages in A20^myel-KO^ and WT, respectively. Each plot features the running enrichment score (*y*-axis) and shows the placement of the ranked differentially expressed genes for the respective transcriptomic signature (*x*-axis).

We next assessed whether type-2 immune activation can suppress M1 macrophage polarization and type-1 immunity upon A20 deficiency and evaluated the sensitivity of A20^myel-KO^ mice to an intestinal parasitic worm (helminth) infection since helminth clearance critically depends on type-2 immunity (27). For this purpose, adult A20^myel-KO^ and WT littermate control mice were inoculated with 200 embryonated *T. muris* eggs by oral gavage and were sacrificed for analysis 21 days later (**Figure 2A**). While WT mice induce robust type-2 immune responses and clear *T. muris* from the intestine to re-establish homeostasis within 21 days (28), A20^myel-KO^ mice show defects in worm clearance and still contain approximately 150–200 *T. muris* parasites in their cecum 21 days post-infection (**Figure 2B**). A20^myel-KO^ mice display persistent infection with *T. muris* and intestinal inflammation characterized by severe immune cell infiltration, tissue damage, and edema (**Figure 2C**). We also show that GATA3 and IL-4, two prototype type-2 immunity markers, are expressed in lower levels in A20^myel-KO^ colon lysates from *T. muris-*infected mice compared with WT littermates (**Figure 2D**). Restimulation of mesenteric lymph node (mLN) cells 21 days post-infection (p.i.) with anti-CD3/CD28 induces the production of type-2 cytokines, such as IL-4, IL-5, and IL-13, in cells derived from WT mice but not in A20^myel-KO^ mLN cells (**Figure 2E**). WT-derived mLN cells are also characterized by higher levels of CD4^+^GATA3^+^ cells (**Figure 2F**). To further evaluate the induction of type-2 immunity in response to *T. muris* infection, we quantified *T. muris-*specific serum IgG1 (type-2-associated) and IgG2c (type-1-associated) immunoglobulins. We found IgG1 to be significantly increased in the serum of WT mice after *T. muris* infection but not in the serum of A20^myel-KO^ mice. In contrast, *T. muris-*specific type-1-associated IgG2c immunoglobulins are increased in A20^myel-KO^ mice but absent in WT mice (**Figure 2G**). We also show increased CD19 and IgG1 levels in WT colon sections after *T. muris* infection, which is not the case in A20^myel-KO^ colon tissue, in line with our findings for serum IgG1 (**Figure 2H**). Immune profiling of colonic lamina propria immune cells after *T. muris* infection (21 days p.i.) confirmed the inability of A20^myel-KO^ mice to mount an effective Th2 response, in contrast to WT littermates. We detected increased immune cell (CD45+) infiltration in A20^myel-KO^ colonic lamina propria, with enhanced levels of neutrophils, monocytes, and macrophages, compared with WT controls (**Figures 3A, B**). In the T-cell compartment, WT mice exhibit significantly elevated levels of CD4^+^GATA3^+^ (Th2) cells, compared with A20^myel-KO^ mice. In contrast, A20^myel-KO^ mice show a skewed Th1/Th17 response, as CD4^+^Tbet^+^ and CD4^+^RORγt^+^ cells are shown to be increased (**Figures 3C, D**). Similar findings are observed in the spleen, confirming a systemic immune response (**Supplementary Figures 4A, B**). It has been reported that the phenotype of the recruited T regulatory cells (Tregs) is indicative of the associated adaptive responses and that Tbet expression in T regulatory cells supports their homeostasis and activity in a type 1-mediated inflammatory context (29). We observe that WT-derived Tregs express higher levels of GATA3, while A20^myel-KO^-derived Tregs express higher levels of Tbet, supporting Th2 and Th1 polarization, respectively (**Supplementary Figures 4D**, **5**). Multiplex imaging on colon sections confirmed the increased number of colonic CD45 cells in WT tissue after infection with *T. muris*; however, immune cell infiltration is even more increased in colons of infected A20^myel-KO^ mice. Similar recruitment patterns are observed for CD11b^+^ myeloid cells and CD64^+^ macrophages (**Figure 3E**). Finally, we performed RNA sequencing on sorted CD11b^+^ cells from the colon of WT and A20^myel-KO^ mice after *T. muris* infection. Samples were isolated and processed 10 days after infection (D10) in order to investigate myeloid cell responses during the peak of infection and anti-helminth immune response. Interestingly, we observe a totally different expression profile for the sorted CD11b^+^ cells of both genotypes (**Supplementary Figure 4C**). In the A20 knockout myeloid cells, we again observe the upregulation of markers related to classical macrophage activation such as *Nos2*, *CD300e* (30), *Bmx* (31), and *Plekhg4* (**Figure 4A**). GO analysis confirms the activation of pathways involved in IFN and TLR4 signaling and IL-1β, IL-12, and IL-22 signaling, which are all related to macrophage polarization toward classical activation (**Figure 4B**). In contrast, in WT mice, we observe the upregulation of markers related to alternative macrophage differentiation such as *CD163* (32), *Nmb* (33), and *Cpeb1* (34). GO analysis supports this notion and indicates upregulation of pathways related to IRF4 targets, PPARγ activation, and IL-4 and GATA3 signaling (**Figures 4A, B**). In order to further evaluate the difference in macrophage polarization, we compared the gene expression profiles of WT and A20^myel-KO^ mice to existing gene signature datasets of macrophage gene expression (21, 35). This confirms that WT mice show enhanced expression of genes involved in alternative macrophage activation (M2-like), whereas A20^myel-KO^ mice show a clear polarization toward classical macrophage activation (M1) upon *T. muris* infection (**Figures 4C, D**), as observed in steady state. Moreover, gene set enrichment analysis (GSEA) confirmed the induction of an M1 phenotype and the downregulation of an M2-like phenotype in A20^myel-KO^ mice compared with WT mice (**Figure 4E**). Collectively, these data clearly indicate that upon *T. muris* infection, WT myeloid cells shift to an alternatively activated state, supporting proper type-2 immune polarization, pathogen clearance, and resolution of inflammation. In contrast, A20-deficient myeloid cells are highly polarized toward classical activation already in steady state, and this immune polarization is not altered upon *T. muris* infection. In conclusion, loss of A20 prevents myeloid cell polarization toward an M2-like state and subsequently impedes proper type-2 immune activation, which prevents parasite clearance and leads to persistent infection and inflammation upon helminth infection.

### 2.3 Type-1 cytokine inhibition promotes *Trichuris muris* resistance in A20^myel-KO^ mice

**Figure 5:**
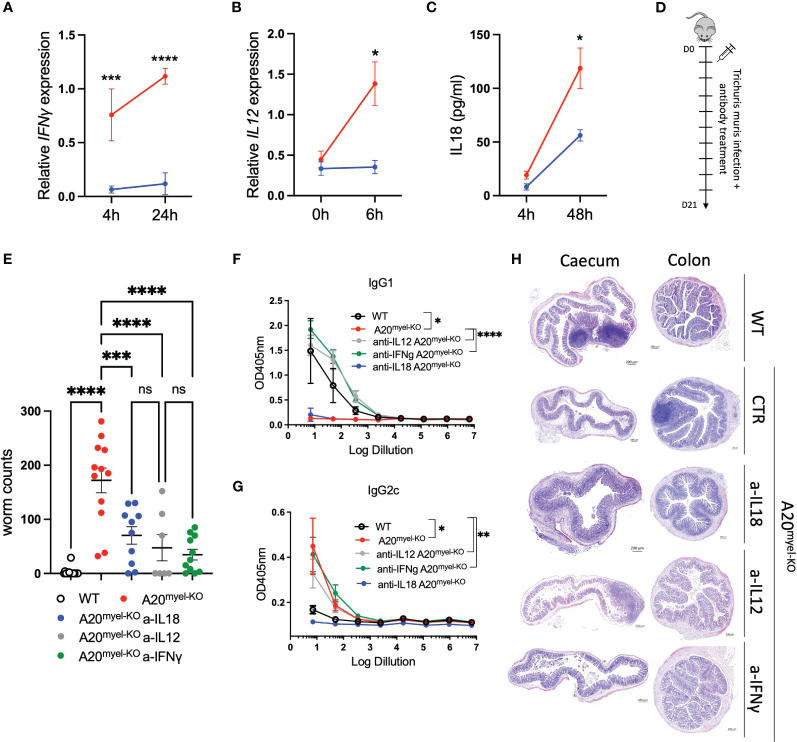
Blocking Th1-related cytokine reverse susceptibility of A20^myel-KO^ to *Trichuris muris*. **(A)** Relative IFN-γ expression in BMDMs derived from WT (blue) and A20^myel-KO^ (red) stimulated with *Trichuris* antigen for 4 h and 24 h. **(B)** Relative expression of IL-12 in WT (blue) and A20^myel-KO^ (red)-derived BMDMs stimulated for 0 h and 6 h with *T. muris* antigen. **(C)** IL-18 levels in the supernatant of WT (blue) and A20^myel-KO^ (red)-derived BMDMs, stimulated with *Trichuris* antigen for 4 h and 48 h. **(D)** Infection and treatment scheme with *T. muris* and neutralizing antibodies against IFN-γ, IL-12, and IL-18. **(E)** Worm counts in the cecum of WT (*n* = 15, open dots), A20^myel-KO^+IgG control antibody (*n* = 12, red), A20^myel-KO^+a-IL-18 (*n* = 10, blue), A20^myel-KO^+a-IL-12 (*n* = 7, gray), and A20^myel-KO^+a-IFN-γ (*n* = 11, green) 21 days after infection with *T. muris*. The graph represents the combined data of three independent experiments. **(F)** ELISA for IgG1 and **(G)** IgG2c levels in the blood serum of WT and A20^myel-KO^ mice treated as in **(B)**. Significance refers to log dilution 1. **(H)** Representative images of AB/PAS staining of cecum and colon sections of WT and A20^myel-KO^ mice infected with *T. muris* and treated with neutralizing antibodies, as in **(B)**. Scale bars 200 μm. Data are presented as means ± s.e.m. Statistical significance was determined by a two-sided Student’s *t*-test or by one-way ANOVA for multiple comparisons. * p<0,05, ** p<0,01, *** p<0,001, **** p < 0.0001. Ns, Not significant.

The Th1-associated cytokine IFN-γ is a very potent antagonist of type-2 polarization and is induced by upstream myeloid-derived cytokines, including IL-12 and IL-18, which, as mentioned earlier, are elevated in A20^myel-KO^ mice (**Supplementary Figure 2G**). *Ex-vivo* differentiated bone marrow-derived macrophages (BMDMs) of WT and A20^myel-KO^ mice were stimulated with *T. muris* antigens and analyzed at different time points. A20^myel-KO^ BMDMs express significantly higher levels of Th1 polarizing cytokines IL-12 and IFN-γ (**Figures 5A, B**) and secrete increased levels of IL-18 (**Figure 5C**) in the supernatant upon *T. muris* antigen stimulation. In order to evaluate the role of IL-12, IL-18, and IFN-γ in preventing an effective type-2 immune response upon *T. muris* infection in A20^myel-KO^ mice, we assessed the outcome of pharmacological inhibition of these cytokines *in vivo*. To this end, A20^myel-KO^ mice were infected with *T. muris* and i.p. injected with neutralizing antibodies against IFN-γ, IL-12, or IL-18 versus isotype control antibody (IgG), every 48 h for a total of 21 days (**Figures 5D, E**). Interestingly, depletion of either one of these three cytokines leads to a significant reduction in the worm counts in the cecum compared with isotype-antibody-treated A20^myel-KO^ mice, with the most potent reduction in anti-IFN-γ-treated mice (**Figure 5E**). We further determined the levels of IgG1 and IgG2c antibodies, as immunoglobulin markers of Th2 and Th1 responses, respectively. We observed that anti-IFN-γ and anti-IL-12 treatment had the most prominent effect in inducing a Th2-associated IgG1 antibody response, as efficient as in WT mice, although all A20^myel-KO^ mice retain increased levels of IgG2c (**Figures 5F, G**). Histological analysis confirms that inflammation is reduced in the colon of A20^myel-KO^ antibody-treated groups (**Figure 5H**). In conclusion, we show that A20^myel-KO^ macrophages overproduce type-1 polarizing cytokines upon recognition of *T. muris* antigens and that neutralization of these specific cytokines *in vivo* can overcome Th1 dominance and improve worm clearance in A20^myel-KO^ mice.

### 2.4 A20^myel-KO^ mice have improved resistance to *Salmonella typhimurium* infection

**Figure 6:**
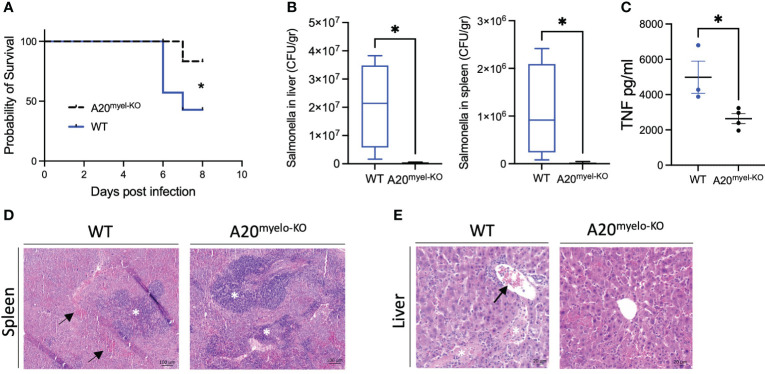
A20^myel-KO^ mice are protected against *Salmonella typhimurium* infection. **(A)** Survival curve of WT (*n* = 7) and A20^myel-KO^ (*n* = 6) mice during 8 days of infection with *S. typhimurium*. Statistical analysis is performed using the Gehan–Breslow–Wilcoxon test. **(B)** CFU counts of retrieved *S. typhimurium* from the liver and spleen of infected WT (*n* = 4) and A20^myel-KO^ (*n* = 5) 8 days post-infection. **(C)** ELISA for TNF levels in the blood serum of WT (*n* = 4) and A20^myel-KO^ (*n* = 5) 8 days post-infection. Data are presented as means ± s.e.m. Statistical significance was determined by a two-sided Student’s *t*-test. **(D)** Representative images of H&E staining of spleens of infected WT and A20^myel-KO^ mice. The black arrows indicate hematopoiesis (red pulp) and the white asterisks indicate the lymphoid tissue (white pulp). Scale bar 100 μm. **(E)** Representative images of H&E staining of the liver of infected WT and A20^myel-KO^ mice. The black arrow indicates immune cell infiltration and the white asterisks indicate the necrotic areas. Scale bar 20 μm. * p<0,05.

Given the strong Th1 immune profile of A20^myel-KO^ mice, we finally evaluated the sensitivity of A20^myel-KO^ mice and WT littermate controls to infection with a virulent strain of *Salmonella enterica* serovar *typhimurium*, as a model for type-1-dependent bacterial infection. WT and A20^myel-KO^ mice were infected by oral gavage with 10^7^ CFU/mouse *S. typhimurium* for 8 days, and disease progression was assessed. A20^myel-KO^ mice show increased resistance against *S. typhimurium* infection compared with WT littermates, as they show increased survival (**Figure 6A**). Moreover, A20^myel-KO^ mice are able to restrict bacterial systemic dissemination and inflammation, as confirmed by significantly lower numbers of viable *S. typhimurium* derived from liver and spleen tissue (**Figure 6B**) and by significantly decreased levels of TNF in the serum (**Figure 6C**). Histological analysis of spleen tissue of WT and A20^myel-KO^ mice showed a decrease of the lymphoid tissue (white pulp) over red pulp in WT compared with A20^myel-KO^ spleens (**Figure 6D**). Moreover, the liver of infected WT mice shows increased immune cell infiltration and necrotic areas compared to the liver of A20^myel-KO^ (**Figure 6E**). Although we do not observe differences in body weight and temperature between the two genotypes (**Supplementary Figure 6C**), A20^myel-KO^ mice are able to clear the *Salmonella* infection more effectively compared to WT littermates, which can be attributed to their enhanced type-1 immunity of A20^myel-KO^ mice.

## 3 Discussion

A20 is a potent anti-inflammatory protein and its molecular role in restricting inflammation and cell death has been extensively documented (36). We recently reported that the inflammatory phenotype of A20^myel-KO^ mice is not protected in germ-free conditions and, therefore, does not depend on microbial triggers (37). In this study, we report the critical role of A20 in macrophage polarization and show for the first time that its expression in myeloid cells is indispensable for the induction of effective Th2 adaptive immunity in response to infection with the helminth *T*. *muris*.

The role of A20 in myeloid and T cells has been studied in an array of disease contexts. Deletion of A20 in myeloid cells in mice leads to the spontaneous development of erosive polyarthritis resembling rheumatoid arthritis in humans (13). Follow-up studies have shown that the arthritic phenotype of A20^myel-KO^ mice is caused by increased Nlrp3 inflammasome activation, leading to increased secretion of IL-1β and IL-18 (12). Moreover, A20 was shown to prevent inflammasome-dependent arthritis by inhibiting macrophage necroptosis (9). A20^myel-KO^ mice were also shown to be resistant to diet-induced obesity and insulin resistance due to the expansion of CD206^+^ adipose tissue macrophages (ATMs), although these ATMs have a gene signature indicative of classical pro-inflammatory macrophage activation (38).

**Figure 7:**
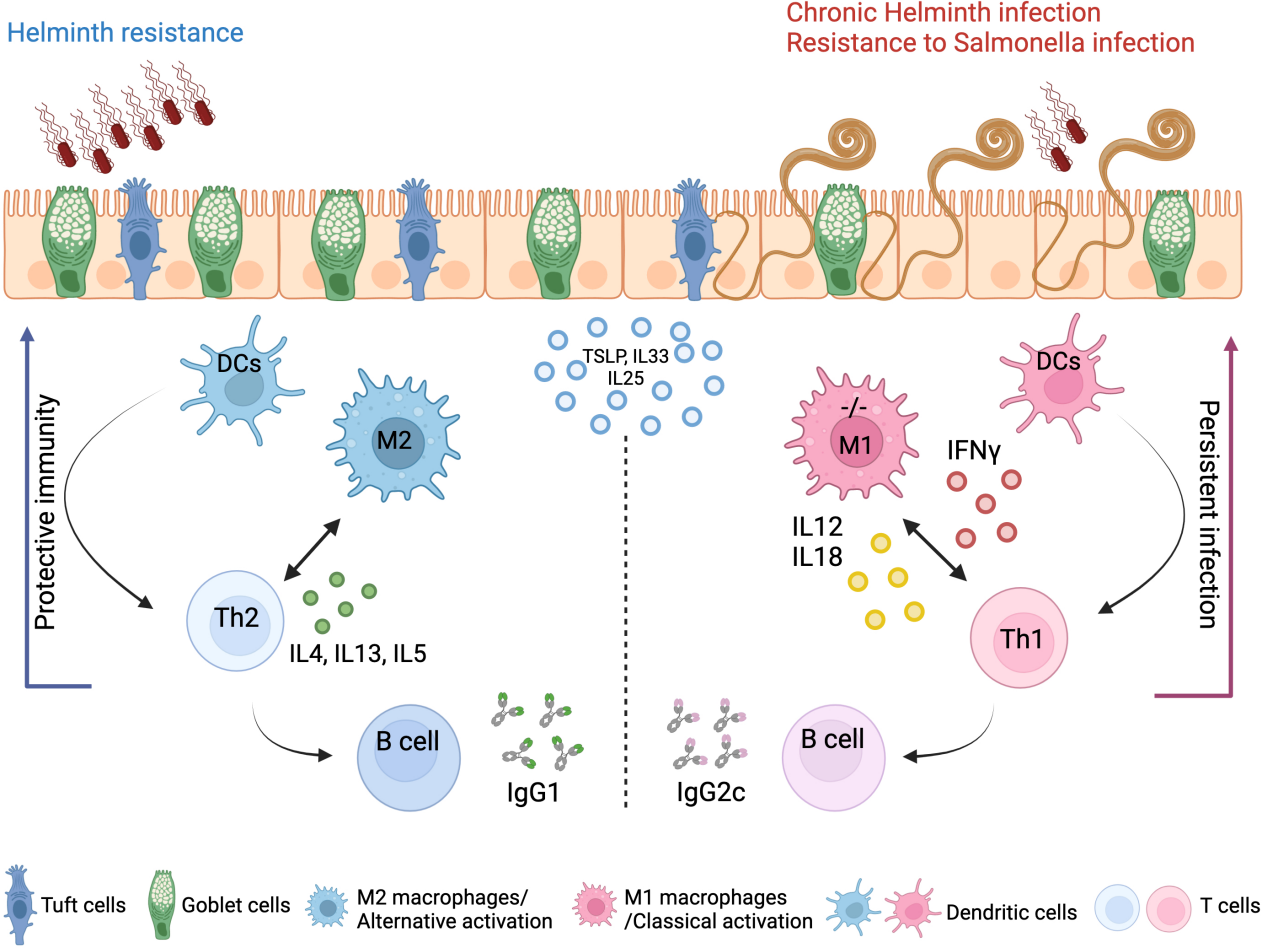
Concluding scheme. The clearance of gastrointestinal helminths depends on type-2 immunity. Helminths interact with and damage intestinal tissue, which leads to the release of intracellular DAMPs and cytokines such as TSLP and IL-33 and IL-25 produced by epithelial cells. These factors activate myeloid cells, which further activate T and B cells to mount effective Th2 responses and the secretion of IL-4, IL-5, and IL-13 cytokines, as well as helminth-specific IgG1 immunoglobulins, leading to effective expulsion of the helminths. Deletion of A20 in the myeloid cells leads to a strong classical macrophage polarization and enhanced secretion of type-1-associated cytokines, including IL-12, IL-18, and IFN-γ, which impede type-2 immune-mediated helminth clearance and promotes persistent infection and intestinal inflammation. Created with BioRender.com.

We show that A20 deletion in myeloid cells predisposes intestinal myeloid cells toward a pro-inflammatory phenotype and M1 polarization in steady state, mediating Th1 skewing in response to *T. muris* infection, which in turn leads to persistent inflammation. In contrast, WT control mice with A20-competent myeloid cells induce effective Th2 immunity and clear the *T. muris* infection (**Figure 7**). Of note, *Gzma*, which we demonstrate to be upregulated in A20^myel-KO^ colon CD11b^+^ cells at steady state, was previously shown to be associated with chronic helminth infection to *Litosomoides sigmodontis*, and granzyme A deletion *in vivo* was shown to promote type-2 immunity (39).

It has been reported that helminths or their secreted molecules, due to their ability to redirect immune responses toward type 2 and induce immunosuppressive mechanisms, can inhibit inflammation and autoimmunity in an array of experimental animal models, including inflammatory bowel disease, multiple sclerosis, and rheumatoid arthritis, but also in humans, as clinical trials to treat autoimmune diseases are ongoing (40, 41). Despite promising data indicating that helminths can suppress various immune disorders, our data show that using live helminths also holds great risks, as dominant genetic factors, including *Tnfaip3* mutations, can instead promote persistent inflammation in response to helminth infection. Regarding the effect of *T. muris* infection on A20^myel-KO^ joint inflammation, we did not observe any reduction in the arthritic phenotype (data not shown). Recently, deletion of A20 in macrophages was reported to suppress IL-33-dependent expansion of lung ILC2s, type-2 cytokine production, and eosinophilia (14). Since ILCs do not play a prominent role during *T. muris* expulsion, in contrast to other parasite infections such as *N. brasiliensis* and *H. polygyrus* infection (42), our data indicate that A20 acts as an essential mediator of alternative macrophage polarization and subsequent adaptive immunity in response to *T. muris* infection. A20 deficiency leads to default classical macrophage activation and type-1 immune polarization. In line with this, enhanced protection of A20^myel-KO^ mice by lethal lung infection with influenza A virus was reported to be mediated by type-1 interferons (i.e., IFN-α/β) (17). While bacterial and viral infections, which depend on type-1 immunity, are cleared more effectively in the absence of myeloid A20, infections with helminths, which depend on type-2 immunity, are compromised. The findings of our study are important for individuals with genetic defects in *TNFAIP3* (or functionally related genes), which are associated with increased susceptibility to various inflammatory and autoimmune pathologies, such as rheumatoid arthritis, systemic lupus erythematosus, multiple sclerosis, and psoriasis (43). Thus, these patients may show reduced resistance against helminth infections but increased protection against Th1-dependent bacterial and viral infections. Taken together, we identify A20 as a critical checkpoint for macrophage and subsequent intestinal mucosal immune polarization and an essential mediator of type-2 immunity in the gut during helminth infection.

## 4 Methods

### 4.1 Animals

A20^myel-KO^ mice, with a myeloid-cell-specific deletion (lysozyme M-Cre) of the A20/Tnfaip3 gene, were previously described (13). Mice were housed in individually ventilated cages at the specific pathogen-free animal facility of the Medical Research Building II at Ghent University Hospital. For our experiments, both males and females were used, evenly distributed over the experimental groups, as we observe no difference in response to *T. muris* and *S. typhimurium* between both sexes. All mice experiments were conducted according to the national Belgian Law (14/08/1986 and 22/12/2003, Belgian Royal Decree 06/04/2010) and European (EU Directives 2010/63/EU, 86/609/EEG) animal regulations.

### 4.2 Propagation of *Trichuris muris* eggs

Six- to eight-week-old RaG2^−/−^ mice were infected with 200 *Trichuris* eggs by oral gavage. After 32–35 days, the mice were sacrificed, and the cecum and proximal colon were isolated. The cecum and colon were cut open to expose the lumen, and the worms were placed in a 10-cm Petri dish containing PBS and, with the help of forceps, were shaken gently to remove feces. The cecum was placed into another Petri dish containing Dulbecco’s modified Eagle medium (DMEM) with 500 U/ml of penicillin and 500 μg/ml of streptomycin (P/S), and by using smooth curved forceps, the worms were gently pulled out and placed in a well of a 6-well plate containing 5 ml of DMEM + P/S. This process was repeated for all the infected mice. The plate was placed in a plastic container with a damp paper towel to maintain humidity and incubated at 37°C for 4 h. After 4 h, the worms were removed using smooth curved forceps and split into two new wells of the six-well plate containing 5 ml of DMEM + P/S. The content of the original well was harvested and placed in a 15-ml falcon tube and spun at 3,000 rpm, and the was supernatant removed and reserved as a 4-h *T. muris* excretory and secretory antigen. This supernatant was used for mLN restimulation and BMDM treatment. The egg-containing pellet was resuspended in autoclaved tap water. The 6-well plate was placed back at 37°C overnight. Thereafter, the worms were removed, and the content of the wells was harvested into a falcon tube and spun at 3,000 rpm for 5 min. The supernatant was collected and reserved as a 24 h *T. muris* excretory and secretory antigen. This supernatant was used for *Trichuris* Ag-specific antibody isotype ELISAs. The remaining egg-containing pellet was resuspended in autoclaved tap water, and the eggs were embryonated at room temperature prior to storage at 4°C for future use.

### 4.3 Infection with *Trichuris muris* helminth

Infection with *T. muris* was performed by oral gavage of 200 embryonated *T. muris* eggs/mouse to 12-week-old WT and A20^myel-KO^ mice. After 21 days, the mice were sacrificed by cervical dislocation for further analysis. For the cytokine neutralization experiments, A20^myel-KO^ mice were infected with *T. muris* eggs as described earlier and treated with antibodies against IFN-γ, IL-12, and IL-18 (BioXcell, Lebanon, USA,, #BE0237) (100 μg). The antibodies were administered i.p. every second day for 21 days in total [IFN-γ (1 mg), IL-12 (1 mg), IL-18 (100 μg)]. Antibodies against IFN-γ and IL-12 were kindly provided by Dr. Louis Boon.

### 4.4 Infection with Salmonella typhimurium

For infection, we used *Salmonella typhimurium*, virulent strain SL1344. Culture: *Salmonella* from glycerol stock was grown in LB for 1 h at 37°C. Next, the liquid culture was plated and grown overnight (ON) on blood agar plates at 37°C to acquire single colonies. The day before infection (D-1), a) a colony of *Salmonella* from the plate was inoculated in 5 ml of LB and grown ON at 37°C in a shaking incubator at 180 rpm and b) the mice were treated with streptomycin to improve colonization efficiency (25 mg/mouse). On the day of infection (D0), food and water were removed from the cage for 4 h. The ON liquid culture was diluted 100× and 500× in LB and grown for 3 h at 37°C. OD600 was measured to determine the concentration and make the appropriate dilutions so that mice were infected with 10^7^ CFU *Salmonella*/200 μl. Water and food were returned to the mice and the animals were monitored for 8 days for body weight change, temperature, and condition. In the end, the mice were sacrificed, and the organs were processed for further analysis. To evaluate bacterial recovery in the spleen, colon, and liver, we used supplemented MacConkey plates [MacConkey agar (CM0007B; Oxoid, Hampshire, United Kingdom), 6.8% sodium thiosulfate (Sigma-Aldrich), 0.8% ferric ammonium citrate (Sigma-Aldrich)].

### 4.5 Flow cytometry

Isolation of colonic lamina propria of WT and A20^myel-KO^ mice was performed as described previously (44). The isolated cells were used for extracellular and intracellular staining for representative markers of T and myeloid cells, in two separated panels (1 × 10^6^ cells per sample) and then analyzed by flow cytometry using a five-laser BD LSRFortessa analyzer. For the analysis of the T-cell compartment, the cells were stained with 7-AAD (1/1,000) for live/dead separation and then extracellularly with the following antibodies: anti-CD3 APC (eBioscience, San Diego, USA; 17-0031-83; 1/100), anti-CD4 APC-Cy7 (BD, New Jersey, USA; 552051; 1/200), and anti-CD8 V500 (BD; 560776; 1/100). The cells were then fixed/permeabilized using the Foxp3 Transcription Factor Staining Buffer Set (eBioscience; 00-5523-00). For the intracellular staining, the following antibodies were used: anti-Foxp3 Alexa Fluor 488 (eBioscience; 53-5773-82; 1/100), anti-T-bet PE-Cy7 (eBioscience; 25-5825-82; 1/100), anti-RORγt BV421 (BD; 562894; 1/100), and anti-GATA3 PE (eBioscience; 12-9966-42; 1/100). For the analysis of the myeloid compartment, the cells were stained with Fixable Viability Dye eFluor 506 (eBioscience; 65-0866-14; 1/300) for live/dead separation and then only extracellularly with the following antibodies: CD19 (eBioscience; 15-0193-82; 1/400), CD3 (eBioscience; 15-0031-82; 1/200), NK1.1 (BioLegend; 108716; 1/200)/PE-Cy5, anti-CD45 Alexa Fluor 700 (eBioscience; 56-0451-82; 1/800), anti-Ly6G PercpCy5.5 (BD; 560602; 1/200), anti-Ly6C APC (eBioscience; 17-5932-80; 1/200), or anti-GR-1 where mentioned (eBioscience; 45-5931-80; 1/200), anti-Siglec-F BUV395 (BD; 740280; 1/200), anti-CD11b BV605 (BD; 563015; 1/600), anti-CD64 BV711 (BioLegend; 139311; 1/100), anti-F4/80 Biotin (eBioscience; 13-4801-82; 1/100), Streptavidin BV421 (BioLegend; 405226; 1/1,000), anti-CD11c PE-eFluor 610 (eBioscience; 61-0114-82; 1/300), and anti-MHC Class II APC-eFluor 780 (eBioscience; 47-5321-80; 1/800). The flow cytometry data were analyzed using FlowJo software and a sequential gating strategy. The graphs showing the percentages of myeloid cells represent the proportions of the parent population. In the tSNE plots for T cells, 10,000 CD3^+^ cells/genotype were exported, and in total, 20,000 cells were analyzed. In the tSNE plots for myeloid cells, 20,000 CD45^+^ cells (lineage excluded: CD19^+^, Nk1.1^+^, and CD3^+^ fraction)/genotype were exported, and in total, 40,000 cells were analyzed. The samples were then concatenated and analyzed with the tSNE plugin of FlowJo (10.6.1) (iterations = 1,000; perplexity = 30). Following dimensional reduction, coordinates for each tSNE dimension (i.e., tSNE1 and tSNE2) in the two-dimensional plots were determined and integrated as novel parameters. The gating strategies for myeloid and T-cell analysis are included in **Supplementary Figures 2H** and **3**.

For the sorting of CD11b^+^ cells from WT and A20^myel-KO^ mice, the cells were stained with anti-CD11b BV605 (BD; 563015; 1/600) and CD19 (eBioscience; 15-0193-82; 1/400), CD3 (eBioscience; 15-0031-82; 1/200), and NK1.1 (BioLegend; 108716; 1/200)/PE-Cy5 as lineage markers (**Supplementary Figures 6A, B**).

### 4.6 Isolation and culture of mesenteric lymph nodes

The mesenteric lymph nodes of WT and A20^myel-KO^ mice were harvested post-mortem. The tissue was dissociated with a plunger through a 70-μm filter. mLN cells were resuspended in RPMI 1640 medium supplemented with fetal calf serum and penicillin/streptomycin and treated with 3 μg/ml of a-CD3 and 5 μg/ml of a-CD28, both soluble for 48 h. After culture, the cells were processed for RNA isolation, cDNA synthesis, and quantitative PCR, and the supernatants were used for cytokine measurement.

### 4.7 *Ex vivo* differentiation of BMDMs

Bone marrow was isolated from the tibia and fibula bones of WT and A20^myel-KO^ mice. The samples were treated with ACK for 3 min at room temperature to remove red blood cells. The cells were then cultured in Petri dishes in 10 ml of RPMI 1640 medium containing 10% fetal calf serum, penicillin/streptomycin, and 40 ng/ml of m-CSF for a total of 7 days. On day 3 (D3), the cells were supplemented with 1 ml of 400 ng/ml m-CSF. On D5, the medium was replaced with a fresh solution containing 40 ng/ml of m-CSF. On D7, the differentiated cells were replated and cultured with 4 h *T. muris* antigen for different time points, i.e., 30 min, 1 h, 2 h, 4 h, 6 h, 24 h, 48 h, and 72 h. At the end of the treatment, the cells were processed for RNA isolation, cDNA synthesis, and quantitative PCR, and the supernatants were used for cytokine measurement.

### 4.8 Quantitative polymerase chain reaction

Tissues and cells were lysed using RLT and β-mercaptoethanol and TissueLyser II (Qiagen, Tegelen, Netherlands, for tissues). Total RNA was isolated using the RNeasy Mini Kit (Qiagen), according to the manufacturer’s instructions. The synthesis of cDNA was performed using QuantiTect^®^ Reverse Transcription Kit (Qiagen). For qPCR, SensiFAST SYBR No-ROX (Bioline, London, United Kingdom) and specific primers for GATA3 (Fw GGCAGAAAGCAAAATGTTTGCT; Rev TGAGTCTGAATGGCTTATTCACAAAT), IL-4 (Fw TGGACTCATTCATGGTGCAG; Rev AACATGGGAAAACTCCATGC), IFN-γ (Fw TCTGGAGGAACTGGCAAAAG; Rev GCTGATGGCCTGATTGTCTT), and IL-12 (Fw CCTGGGTGAGCCGACAGAAGC; Rev CCACTCCTGGAACCTAAGCAC) were used on LightCycler 480 (Roche, Basel, Switzerland). The reactions were performed in triplicates, and the results were analyzed with qbase+ software. As housekeeping genes, *GAPDH*, *Actb*, and *Ubc* were used.

### 4.9 ELISA for IgG1 and IgG2c levels in blood serum

The plates for the ELISA were coated overnight at 4°C with diluted *Trichuris* antigen in 0.05 M of carbonate bicarbonate buffer. After washing 5× with PBST 0.05%, the plate was incubated with blocking buffer (3% BSA+PBST 0.05%). The diluted serum was then added to the plate and incubated for 90 min at room temperature (RT). Later, the plate was washed 5× with PBST 0.05% and incubated with biotinylated antibody G1 (Serotec MCA336B) or G2A (BD Bioscience 553388) for 60 min at RT. After five washes with PBST 0.05%, the plate was incubated with Streptavidin-POD conjugate (Roche, 11089153001) for 1 h at RT. Next, the peroxidase substrate ABTS (2,2′-azinobis [3-ethylbenzothiazoline-6-sulfonic acid]-diammonium salt, Sigma-Aldrich, A1888) was added, and the absorbance was determined at 405 nm, with reference to 490 nm.

### 4.10 Quantification of cytokines

Cytokine levels were determined in mouse blood serum and in supernatants of *ex-vivo* cultured mesenteric lymph nodes and bone marrow-derived macrophages. Mouse blood was collected post-mortem by cardiac puncture and stored in clot activator tubes containing serum separating silica particles.

IL-4 (171G5005M), IL-5 (171G5006M), and IL-13 (171G5012M) were quantified using the Bio-Plex Pro kit (Bio-Rad, California, United States) on the Bio-Plex 200 system (Bio-Rad) according to the manufacturer’s instructions. IFN-γ, IL-12, and IL-18 were quantified using ELISA (R&D Systems, Minneapolis, United States).

### 4.11 Histology

Mouse colon and cecum tissues were fixed in formalin (10%; neutral buffered; Sigma-Aldrich, Missouri, United States). Samples were dehydrated, embedded in paraffin, sectioned at 5 μm, stained with hematoxylin and eosin, and examined by light microscopy. For combined Alcian Blue and periodic acid Schiff staining, dewaxed sections were dehydrated and incubated in Alcian Blue for 20 min. The sections were subsequently washed with PBS and treated with 1% periodic acid for 15 min followed by incubation in Schiff’s reagent for 15 min. Sections were counterstained with Mayer’s hematoxylin for 30 s, washed, and dehydrated before mounting with DePeX.

### 4.12 MACSima platform

The following samples were prepared for MACSima imaging: colon—WT colon steady state, A20^myel-KO^ colon steady state, WT colon *T. muris*, A20^myel-KO^ colon *T. muris*. The tissues were mounted in OCT compound, and sections of 8 μm thickness were prepared with a cryostat. The sections were later fixed with acetone at −20°C for 10 min and washed 3× with PBS, and staining proceeded. Thirty-two markers in total were assessed with the MACSima technology which were coupled to PE or FITC fluorophores. According to the basic principle of the technology, validated antibodies by Miltenyi enable the identification of multiple markers at the same time. The MACSima imaging system acquires fluorescence images of two markers/cycle of the desired predefined ROIs. To complete the cycle, the fluorescence signal is erased, and the process restarts automatically. The images were later analyzed with MACSiQView software.

### 4.13 Bulk RNA sequencing and analysis

Sorted CD11b^+^ cells from the colon lamina propria and from the (ankle and knee) joint synovium were analyzed with bulk RNA sequencing using a QuantSeq mRNA Library Prep on 37 samples and sequencing on the NextSeq500 SR-75 by NXTGNT (Faculty of Pharmaceutical Sciences, Ghent University). The quality of the raw data was checked using FastQC (version 0.11.9) and MultiQC (version 1.12). The trimmed data (Trimmomatic version 0.39) were mapped to the mouse reference genome (Assembly: GRCm39, GCA_000001635.9) using STAR (version 2.7.10a). BAM files were created using Samtools (version 1.9) and counted with featureCounts from the Subread package (version 2.0.3).

The quality of the experimental design was assessed using PCA and sample-to-sample distance to determine outlier samples. DESeq2 (version 1.38.2) was used to perform differential expression analysis. *P*-values were adjusted for multiple testing by DESeq2 using Benjamini–Hochberg correction. Genes were labeled as differentially expressed when the adjusted *P*-value <0.05. Functional enrichment was performed using the fgsea (v1.24.0) package to find enriched pathways. A pre-ranked gene list was generated by ordering the shrunk differential expression results according to log2FC. Enrichment results were filtered using a normalized enrichment score (NES) cutoff >1.

Gene scoring was performed by comparing the expression data of our dataset with published expression data for *in-vitro* M1 classically activated (IFN-γ+LPS) macrophages and M2 alternatively activated (IL-4) macrophages described by Orecchioni M. et al. (21) This gene list consists of 710 genes identified to be upregulated in classically activate macrophages and 806 genes to be upregulated in alternatively activated macrophages. The RNA sequencing datasets are available at public repositories (**Supplementary Data**).

### 4.14 Statistical analysis

Data were analyzed using GraphPad Prism 9.4.1 software. The results are expressed as means ± s.e.m. We used the unpaired Student’s *t*-test for two-parameter comparisons or one-way ANOVA for multiple comparisons. Differences with *P <*0.05 were considered statistically significant and denoted as *P < 0.05, **P < 0.01, ***P < 0.001, ****P < 0.0001.

## Data availability statement

The original codes presented in the study are publicly available.The data can be found in the link below: https://github.com/VereeckeLab/Myeloid-A20-is-critical-for-type-2-immune-mediated-helminth-resistanceThe raw data and count matrix are available in the following link:https://zenodo.org/records/10926657.

## Ethics statement

The animal study was approved by the Inflammation Research Center (IRC) and the Animal Ethics Committee of Ghent University. The study was conducted in accordance with the local legislation andinstitutional requirements.

## Author contributions

IP: Funding acquisition, Writing – review & editing, Writing – original draft, Visualization, Validation, Supervision, Methodology, Investigation, Formal analysis, Data curation, Conceptualization. MT: Validation, Methodology, Investigation, Writing – review & editing, Formal analysis, Data curation. MC: Validation, Methodology, Writing – review & editing, Software, Formal analysis, Data curation. GB: Investigation, Formal analysis, Writing – review & editing, Validation, Methodology, Data curation. LB: Investigation, Conceptualization, Writing – review & editing, Resources. TM: Validation, Writing – review & editing, Software, Formal analysis, Data curation. FN: Writing – review & editing, Software, Formal analysis, Data curation. AW: Conceptualization, Writing – review & editing, Resources, Methodology. RG: Investigation, Writing – review & editing, Resources, Methodology. DE: Resources, Writing – review & editing, Supervision, Methodology. GL: Resources, Writing – review & editing, Supervision, Methodology. LV: Validation, Investigation, Writing – review & editing, Writing – original draft, Visualization, Supervision, Resources, Project administration, Methodology, Funding acquisition, Conceptualization.

## Supplementary material

Supplementary Figure 1
**(A)** Representative images of H&E staining of ankle joint of WT and A20^myel-KO^ mice, 14 weeks-old. Arrows indicate the sites of immune cell infiltration. **(B)** Representative images of AB/PAS staining of colon sections of WT and A20^myel-KO^ mice, 14 weeks-old. **(C)** Representative macroscopic images of WT and A20^myel-KO^ spleens after dissection at 14 weeks-old. Flow cytometry analysis of spleen of WT (n=6) and A20^myel-KO^ (n=6) mice for the myeloid compartment at steady state, including CD11b+ **(D, E)**, Monocytes **(F, G)**, Neutrophils and Eosinophils (H-J). (K) Flow cytometry for T cell analysis in the spleen of WT (n=6) and A20^myel-KO^ (n=6) including CD4+RORγt+, CD4+Foxp3+ (presented as percentage of CD4+ cells), CD4+ and CD8+ (presented as percentage of CD3+ cells). Data are presented as means ± s.e.m. Statistical significance was determined by a two-sided Student’s t-test.

Supplementary Figure 2
**(A)** Bulk RNA sequencing volcano plots presenting the differentially expressed genes in CD11b+ sorted cells of WT (n=5, blue) and A20^myel-KO^ (n=5, magenta) joint synovium samples. **(B)** Heatmap showing the top 25 M1 markers to be upregulated in A20^myel-KO^ synovium compared to WT. **(C)** Gene ontology pathway analysis showing the positively regulated pathways in A20^meyl-KO^ synovium CD11b+ cells. **(D)** Gene scoring analysis using an M1 signature to compare the expression in WT and A20^myel-KO^ synovium. **(E)** Bulk RNA sequencing and clustering of WT (n=5) and A20^myel-KO^ (n=5) colon and **(F)** of WT (n=4) and A20^myel-KO^ (n=5) joint samples at steady state on PCA plots according to gene expression. **(G)** Relative IL18 expression in BMDMs of WT (n=4) and A20^myel-KO^ (n=4) in steady state condition. Data are presented as means ± s.e.m. Statistical significance was determined by two-sided Student’s t-test. **(H)** Gating strategy for flow cytometry analysis of myeloid cells in colon and spleen.

Supplementary Figure 3 Gating strategy for flow cytometry analysis data for T cells in colon and spleen. Details for the immune markers analysed and fluorophores used are included in materials and methods section.

Supplementary Figure 4 Flow cytometry analysis in the spleen of WT (n=6) and A20^myel-KO^ (n=4) upon *Trichuris muris* infection including **(A)** total CD11b+, Monocytes, Neutrophils and Dendritic cells for the Myeloid compartment. **(B)** Analysis of the T cell compartment includes CD4+, CD8+ (presented as percentage of CD3+ cells), CD4+Tbet+, CD4+RORgt+, CD4+Foxp3+ and CD4+GATA3+ levels (presented as percentage of CD4+ cells). **(C)** Bulk RNA sequencing and clustering of WT colon at steady state (n=5, blue triangle) and upon *Trichuris muris* (n=4, blue square) versus A20^myel-KO^ colon at steady state (n=5, red triangles) and upon *Trichuris muris* infection (n=4, red squares) combined on a PCA plot according to gene expression. **(D)** GATA3, Tbet and RORγt expression within colonic T regulatory (percentage of CD4+Foxp3+) cell population in WT (n=6) and A20^myel-KO^ (n=4) upon *Trichuris muris* infection.

Supplementary Figure 5 FlowSOM analysis of WT (n=4) and A20^myel-KO^ (n=4) upon *Trichuris muris* infection for T cell composition. The data are visualized in minimal spanning trees and according to the parameter’s expression levels, Flowsom organizes the branches of the tree (cell clusters) in 8 different metaclusters (0-7). The parameters analysed are colored inside every circle as following: Tbet=magenta, GATA3=purple, CD4=light blue, CD8=green, RORγt=yellow and Foxp3=orange. The height of each part indicates the expression intensity: the higher the part in the circle (node), the higher the expression of the marker is. The size of the circles is also indicative of the abundance of the representative cell cluster. (A) In WT mice upon *Trichuris infection* we observe an abundance of the cell cluster which is represented by circles that contain higher purple parts (GATA3). In contrast, A20^myel-KO^ mice show abundance of the cell cluster which is represented by circles that contain higher magenta parts (Tbet). We also observe increase of the circles contain higher yellow parts (RORγt). Tregs are represented by orange parts and appear as a heterogenous population separated in 2 branches, with higher expression of RORγt (yellow), Tbet (magenta) and GATA3 (purple). The samples were exported and concatenated and a total of 10.000 CD3+ cells/genotype is analysed. The parameters applied for the Flowsom analysis are the following: Number of metaclusters=8, SOM (self-organizing map) grid size 10x10, Node scale=300%. FlowJo plugin 3.0.18 (45). The different metaclusters present the differential and combinatorial expression of the analysed parameters. 5200 CD3+ cells were extracted and concatenated/genotype (in total 10400 cells) to perform Flowsom analysis.

Supplementary Figure 6 Fluorescence-activated cell sorting of CD11b+ from **(A)** joint synovium and **(B)** colon lamina propria of WT and A20^myel-KO^ mice for bulk RNA sequencing. Representative plots of sorted and re-sorted DC11b+ cells to ensure the purity of the sorted population. **(C)** Graphs presenting the % body weight loss (left) and % body temperature change (right) in WT (blue) and A20^myel-KO^ (black dotted) mice upon infection with *Salmonella typhimurium*. **(D)** Representative H&E staining of colon section of WT and A20^myel-KO^ on day 8 p.i. **(E)** CFU counts of retrieved *Salmonella typhimurium* from stool samples of infected WT (n=4) and A20^myel-KO^ (n=5). **(F)** Colon length of infected WT (n=3) and A20^myel-KO^ (n=5) 8 days post infection. **(G)** McConkey plates showing *Salmonella typhimurium* colony growth in WT (n=2/plate) and A20^myel-KO^ (n=2/plate) from liver samples on day 8 p.i. The samples are serially diluted (1/10) left to right (D1->D3).
